# Prescription factors influencing baloxavir prescription during the 2018/2019 and 2019/2020 seasons: a administrative database study in Japan

**DOI:** 10.1186/s40780-023-00274-1

**Published:** 2023-02-01

**Authors:** Naomi Fujiwara, Takashi Fujiwara, Yuya Ise

**Affiliations:** 1 Department of Pharmacy, Nippon Medical School Hospital, Sendagi 1-1-5, Bunkyo-Ku, Tokyo, 113-8602 Japan; 2 Department of Public Health Research, Kurashiki Clinical Research Institute, Miwa 1-1-1, Kurashiki City, Okayama, 710-8602 Japan

**Keywords:** Baloxavir, Influenza, Administrative claims

## Abstract

**Background:**

We aimed to evaluate the factors associated with baloxavir prescription in Japanese hospitals using a health insurance claim-based database (MDV analyzer), during the 2018/2019 and 2019/2020 influenza seasons. The MDV analyzer contains anonymized claims data from approximately 420 Diagnosis Procedure Combination hospitals, and does not contain data from clinics.

**Methods:**

Data were collected for influenza patients treated with anti-influenza drugs during the 2018/2019 and 2019/2020 influenza seasons. Multivariate analysis was used to identify factors associated with baloxavir prescription.

**Results:**

During the study period, 322,063 influenza patients were included for analyses. In multivariate analysis, children, female sex, inpatient, hospital bed capacity, and private hospitals were negatively associated with baloxavir prescription. Compared to elderly patients, the adjusted odds ratio (OR) for baloxavir prescription was 0.612 (95% confidence interval (CI), 0.587–0.637) in children aged 6–11 years, and 0.119 (95% CI, 0.111–0.128) in children aged 0–5 years. Compared to small hospitals (bed capacity, 20–299), the adjusted OR for baloxavir prescription was 0.559 (95% CI, 0.540–0.578) in large hospitals (bed capacity, ≥ 500).

**Conclusion:**

Children, female sex, inpatient, hospital bed capacity, and private hospitals were negatively associated with baloxavir prescription.

**Supplementary Information:**

The online version contains supplementary material available at 10.1186/s40780-023-00274-1.

## Background

Seasonal influenza is an infectious disease that is usually characterized by fever, sore throat, myalgia, headache, cough, and fatigue. Approximately 10–20 million people are affected annually by influenza in Japan, which usually peaks between November and March [1]. Most cases of seasonal influenza resolve within a week without treatment; however, it may cause severe illnesses in high-risk patient groups such as children, older adults, and patients with comorbidities; therefore, neuraminidase inhibitors are recommended for high-risk patients [2]. Of note, in Japan, neuraminidase inhibitors are also recommended for low-risk patients to shorten the duration of fever and other symptoms [3].

Baloxavir, a cap-dependent endonuclease inhibitor, was approved for treating influenza in Japan in February 2018. Clinical trials have shown that an oral single-dose baloxavir is similar to oseltamivir in terms of effectiveness [4]. Oseltamivir is the most common neuraminidase inhibitors, and is widely used in Japan. Oseltamivir usually requires a five-day oral treatment regimen, although non-compliance to the drug is a major concern for reduced efficacy and emergence of resistant strains [5–7]. Laninamivir is a long-acting neuraminidase inhibitors, and is administered by nasal inhalation as a single dose. Though laninamivir treatment is completed in one day, it is not acceptable for people who cannot inhale well, like children and patients with respiratory disease.

An online survey found that patients prefer oral, short-term, less frequent (once daily) anti-influenza drugs [8]. Indeed, baloxavir was prescribed for 43% of patients with influenza diagnosed in the 2018/2019 season [9]. However, no study evaluated the factors associated with baloxavir prescription using real-world prescription data. In this study, we aimed to evaluate the factors associated with baloxavir prescription in Japan using a large claim-based database during the 2018/2019 and 2019/2020 influenza seasons.

## Methods

### Data source and eligible criteria

This cross-sectional study was conducted using an MDV analyzer (Medical Data Vision Co., Ltd., Tokyo, Japan). The MDV analyzer contains anonymized claims data from approximately 420 diagnosis-procedure combination (DPC) hospitals. The MDV analyzer does not contain data from clinics. After selecting the target International Classification of Diseases version 10 (ICD-10) codes, drugs, and months, the MDV analyzer provides the number of cases by age and sex that meet the target conditions. In this study, we included patients who were diagnosed with the ICD-10 codes of influenza (J09 [Influenza due to certain identified influenza viruses], J10 [Influenza due to other identified influenza virus], and J11 [Influenza due to unidentified influenza virus]) and treated with anti-influenza drugs including baloxavir, oseltamivir, zanamivir, peramivir, and laninamivir during the 2018/2019 season (from November 2018 to March 2019) and 2019/2020 season (from November 2019 to March 2020). The dataset for this study was extracted from the MDV analyzer on March 13, 2021.

### Data collection and outcome

The primary outcome was the baloxavir prescription. The MDV analyzer includes data on age, sex, setting (admitted or outpatient), hospital beds, type of hospital, and drugs. All of these parameters were used in the analysis. We also used the seasons (2018/2019 or 2019/2020 season) as parameters for analysis.

### Statistical analysis

To identify potential predictive factors for baloxavir prescription, we performed univariate Fisher’s exact test and multivariable logistic regression analyses. We used baloxavir prescription as dependent variable (baloxavir versus other anti-influenza drugs [baloxavir, oseltamivir, zanamivir, peramivir, and laninamivir], and others (age groups [≥ 65, 12–64, 6–11, or ≤ 5], sex [male or female], setting [outpatient or inpatient], hospital beds [20–299, 300–499, or ≥ 500], type of hospital [public hospital, private hospital, university hospital], and seasons [2018/2019 or 2019/2020 season]) as independent variable. A *P* value of < 0.05 was considered statistically significant. Statistical analyses were performed using R version 4.0.3 (R Foundation for Statistical Computing, Vienna, Austria) and EZR version 1.61 [10]. This study was approved by the Institutional Review Board of Nippon Medical School Hospital (B-2021–382) and conducted following the Declaration of Helsinki. The requirement for informed consent was waived as the study used anonymized retrospective data.

## Results

During the study periods, 322,063 influenza patients were treated with anti-influenza drugs. The baseline characteristics of the included patients are shown in Table 1. The results of the univariate and multivariate analyses are shown in Table 2. In the multivariate analyses, children, female sex, inpatient, hospital bed capacity, private hospital, and 2019/2020 season were negatively associated with baloxavir prescription.

**Table 1:** Baseline characteristics of the included patients

	Overall (*n* = 322,063)	Baloxavir prescription (*n* = 41,083)	Other antivirals prescription (*n* = 280,980)	Rate of baloxavir prescription
**Age (years)**				
≥ 65	54,041	8,066	45,975	(14.9%)
12–64	176,624	27,532	149,092	(15.6%)
6–11	45,701	4,478	41,223	(9.8%)
≤ 5	45,697	1,007	44,690	(2.2%)
**Sex**				
Male	166,786	21,401	145,385	(12.8%)
Female	155,277	19,682	135,595	(12.7%)
**Setting**				
Outpatient	294,340	39,546	254,794	(13.4%)
Inpatient	27,723	1,537	26,186	(5.5%)
**Hospital beds**				
20–299	98,439	16,367	82,072	(16.6%)
300–499	148,829	17,592	131,237	(11.8%)
≥ 500	74,795	7,124	67,671	(9.5%)
**Type of hospital**				
Public hospital	194,798	23,540	171,258	(12.1%)
Private hospital	117,940	16,564	101,376	(14.0%)
University hospital	9,325	979	8,346	(10.5%)
**Seasons**				
2018/2019 season	198,618	36,471	162,147	(18.4%)
2019/2020 season	123,445	4,612	118,833	(3.7%)

**Table 2:** Univariate and multivariable analyses

	Univariate		Multivariate	
	Odds ratio (95% CI)	*P*-value	Odds ratio (95% CI)	*P*-value
**Age (years)**				
≥ 65	Reference		Reference	
12–64	1.050 (1.020–1.080)	< 0.001	0.946 (0.919–0.975)	< 0.001
6–11	0.619 (0.596–0.644)	< 0.001	0.612 (0.587–0.637)	< 0.001
≤ 5	0.128 (0.120–0.137)	< 0.001	0.119 (0.111–0.128)	< 0.001
**Sex**				
Male	Reference		Reference	
Female	0.986 (0.966–1.010)	0.185	0.949 (0.928–0.969)	< 0.001
**Setting**				
Outpatient	Reference		Reference	
Inpatient	0.378 (0.359–0.399)	< 0.001	0.346 (0.327–0.366)	< 0.001
**Hospital beds**				
20–299	Reference		Reference	
300–499	0.672 (0.657–0.688)	< 0.001	0.727 (0.709–0.745)	< 0.001
≥ 500	0.528 (0.512–0.544)	< 0.001	0.559 (0.540–0.578)	< 0.001
**Type of hospital**				
Public hospital	Reference		Reference	
Private hospital	1.190 (1.160–1.210)	< 0.001	0.882 (0.861–0.904)	< 0.001
University hospital	0.853 (0.798–0.913)	< 0.001	0.966 (0.900–1.040)	0.348
**Seasons**				
2018/2019 season	Reference		Reference	
2019/2020 season	0.173 (0.167–0.178)	< 0.001	0.171 (0.165–0.176)	< 0.001

Main predictors for baloxavir prescription

To confirm these associations, we also conducted multivariate analyses in each season. Multivariate analyses in each season are available in Additional Tables 1 and 2. The change of prescription rate of baloxavir from 2018/2019 to 2019/2020 seasons subgroups is shown in Additional Fig. 1. To identify predictive factors for each anti-influenza drug, we conducted additonal analyses. The results are available in Additional Tables 3–7.

## Discussion

In the multivariable analysis, the adjusted odds ratio for children, season, outpatient, and high bed capacity were the first, second, third, and fourth factors most strongly negatively associated with baloxavir prescription. Additional Table 3–7 shows influencing factors for each anti-influenza drug. Inhaled drugs (laninamivir and zanamivir) were often prescribed for patients aged 12–64, and aged 6–11. Although, oseltamivir was prescribed less often for patients aged 12–64, and aged 6–11. Premivir is intravanously administrated, and was often prescribed in inpatient settings.

Prescribing decisions are influenced by several factors, including physicians' personal preference, cost of treatment, pharmaceutical industries’ marketing and promotion strategies, and patients’ preferences [11]. Baloxavir has been shown to have a similar effectiveness to oseltamivir in a clinical trial [4], and has a high cost; hence, other factors must influence baloxavir prescription. An online survey of adults found that patients prefer oral, short-term, less-frequent (once daily) anti-influenza drugs [8]. Physicians are likely to have been influenced by patients’ preferences, and prescribed baloxavir during the study period. The odds ratio of baloxavir prescription decreased as the number of hospital beds increased. Large hospitals employ more physicians; due to peer pressure, physicians may give priority to the recommendations from academic associations over the patients' preferences [12]. A previous online survey on parents reported that parents also prefer oral, short-term, less-frequent (once daily) anti-influenza drugs for their children [11], although the adjusted odds ratio of baloxavir prescription was lower in children. Pediatricians have a greater tendency to adhere to guidelines compared with physicians working in other medical disciplines [13]. Differences in preferences among clinical departments are likely to have led to the difference in the baloxavir prescription rate according to age.

The 2019/2020 season was negatively associated with baloxavir prescription. Generally, the efficacy of the newly drug is determined by clinical trials (randomized controlled trials), and the effectiveness and adverse events are assessed in real-world settings (cohort studies). In the 2018/2019 season, the effectiveness and adverse events of baloxavir was unclear, because it had just arrived in the market. After the wide use of baloxavir in 2018/2019 season, several cohort studies assess the drug. In 2019 October, National Institute of Infectious Diseases, Japan reported that the prevalence of baloxavir-resistant influenza were 10% or more in pediatric and elderly patients [14]. Therefore in 2019/2020 season, Japan Pediatric Association decided not to recommend the use of baloxavir in pediatric influenza patients [15]. The reduction of baloxavir prescriptions from the 2018/2019 season to the 2019/2020 season would be attributable to statements from academic associations, wide recognition of the side effects, and antimicrobial stewardship programs [15, 16].

This study has certain limitations. We could not assess potential risk factors associated with baloxavir prescription (e.g., comorbidities, and presence of an infection control team). This information could not be accessed through the MDV analyzer. Additionally, the MDV analyzer does not contain data from clinics. In a report from the Ministry of Health, Labor and Welfare of Japan, the prescription rate of baloxavir in 2018/2019 season was 43% among all influenza cases nation widely [9]. However, the rate reported in this study was only 18.2%. The discrepancy might stem from baloxavir being more commonly prescribed in clinics than in hospitals. Therefore, further studies are needed to assess using both hospitals and clinics.

## Conclusion

In conclusion, children, female sex, inpatient, hospital bed capacity, and private hospitals negatively correlates with baloxavir prescription for influenza during the 2018/2019 and 2019/2020 seasons in Japan.

## Supplementary Information

**Additional file 1:** **Additional Table 1.** Multivariate analysis of patient characteristics in 2018/2019 season. **Additional Table 2.** Multivariate analysis of patient characteristics in 2019/2020 season. **Additional Table 3.** Multivariate analysis of patient characteristics in 2018/2019 season. **Additional Table 4.** Multivariate analysis of patient characteristics in 2018/2019 season. **Additional Table 5.** Multivariate analysis of patient characteristics in 2018/2019 season. **Additional Table 6.** Multivariate analysis of patient characteristics in 2018/2019 season. **Additional Table 7.** Multivariate analysis of patient characteristics in 2018/2019 season. **Additional Figure 1.** Baloxavir prescription rate from the 2018/2019 season to the 2019/2020 season.

## Data Availability

The datasets used and/or analyzed during the current study are available from the corresponding author on reasonable request.

## Abbreviations

DPC: Diagnosis-procedure combination
ICD-10: International classification of diseases version 10
