# Supplementary material for: Prescription factors influencing baloxavir prescription during the 2018/2019 and 2019/2020 seasons: a administrative database study in Japan
Source: J Pharm Health Care Sci. 2023 Feb 1;9:3. doi: 10.1186/s40780-023-00274-1 (PMC9890836; doi:10.1186/s40780-023-00274-1)
Supplement: Supplementary file 1 — Additional file 1: Additional Table 1. Multivariate analysis of patient characteristics in 2018/2019 season. Additional Table 2. Multivariate analysis of patient characteristics in 2019/2020 season. Additional Table 3. Multivariate analysis of patient characteristics in 2018/2019 season. Additional Table 4. Multivariate analysis of patient characteristics in 2018/2019 season. Additional Table 5. Multivariate analysis of patient characteristics in 2018/2019 season. Additional Table 6. Multivariate analysis of patient characteristics in 2018/2019 season. Additional Table 7. Multivariate analysis of patient characteristics in 2018/2019 season. Additional Figure 1. Baloxavir prescription rate from the 2018/2019 season to the 2019/2020 season. [file 40780_2023_274_MOESM1_ESM.docx]

# Additional Table 1. Multivariate analysis of patient characteristics in 2018/2019 season

|  | Multivariate analysis | | |
| --- | --- | --- | --- |
|  | Adjusted odds ratio (95% CI) | | P-value |
| **Age groups** |  |  |  |
| ≥ 65 | Ref |  |  |
| 12–64 | 0.960 | (0.930–0.990) | 0.010 |
| 6–11 | 0.692 | (0.663–0.723) | <0.001 |
| ≤5 | 0.128 | (0.120–0.138) | <0.001 |
| **Sex** |  |  |  |
| Male | Ref |  |  |
| Female | 0.950 | (0.929–0.973) | <0.001 |
| **Setting** |  |  |  |
| Outpatient | Ref |  |  |
| Inpatient | 0.348 | (0.327–0.369) | <0.001 |
| **Hospital beds** |  |  |  |
| 20–299 | Ref |  |  |
| 300–499 | 0.699 | (0.681–0.718) | <0.001 |
| ≥ 500 | 0.565 | (0.545–0.585) | <0.001 |
| **Type of hospital** |  |  |  |
| Public hospital | Ref |  |  |
| Private hospital | 0.840 | (0.818–0.861) | <0.001 |
| University hospital | 0.953 | (0.884–1.030) | 0.211 |

# Additional Table 2. Multivariate analysis of patient characteristics in 2019/2020 season

|  | Multivariate analysis | | |
| --- | --- | --- | --- |
|  | Adjusted odds ratio (95% CI) | | P-value |
| **Age groups** |  |  |  |
| ≥ 65 | Ref |  |  |
| 12–64 | 0.821 | (0.755–0.892) | <0.001 |
| 6–11 | 0.230 | (0.201–0.264) | <0.001 |
| ≤5 | 0.052 | (0.039–0.069) | <0.001 |
| **Sex** |  |  |  |
| Male | Ref |  |  |
| Female | 0.938 | (0.884–0.996) | 0.036 |
| **Setting** |  |  |  |
| Outpatient | Ref |  |  |
| Inpatient | 0.353 | (0.296–0.421) | <0.001 |
| **Hospital beds** |  |  |  |
| 20–299 | Ref |  |  |
| 300–499 | 0.928 | (0.869–0.991) | 0.027 |
| ≥ 500 | 0.481 | (0.434–0.535) | <0.001 |
| **Type of hospital** |  |  |  |
| Public hospital | Ref |  |  |
| Private hospital | 1.210 | (1.140–1.290) | <0.001 |
| University hospital | 1.050 | (0.838–1.310) | 0.674 |

# Additional Table 3. Multivariate analysis of patient characteristics in 2018/2019 season

Data source: 2018-Nov, 2018-Dec, 2019-Jan, 2019-Feb, 2019-Mar

Data extraction date: March 28, 2021

Outcome: **Baloxavir** prescription

Variables: Age groups, sex, setting (admitted or outpatient), and hospital beds (20–299, 300–499, or ≥ 500)

|  | Multivariate analysis | | |
| --- | --- | --- | --- |
|  | Adjusted odds ratio (95% CI) | | P-value |
| **Age groups** |  |  |  |
| ≥ 65 | Ref |  |  |
| 12–64 | 0.962 | (0.932–0.992) | 0.014 |
| 6–11 | 0.701 | (0.672–0.732) | <0.001 |
| ≤5 | 0.130 | (0.121–0.140) | <0.001 |
| **Sex** |  |  |  |
| Male | Ref |  |  |
| Female | 0.949 | (0.927–0.971) | <0.001 |
| **Setting** |  |  |  |
| Outpatient | Ref |  |  |
| Inpatient | 0.335 | (0.316–0.356) | <0.001 |
| **Hospital beds** |  |  |  |
| 20–299 | Ref |  |  |
| 300–499 | 0.764 | (0.745–0.784) | <0.001 |
| ≥ 500 | 0.612 | (0.592–0.632) | <0.001 |

# Additional Table 4. Multivariate analysis of patient characteristics in 2018/2019 season

Data source: 2018-Nov, 2018-Dec, 2019-Jan, 2019-Feb, 2019-Mar

Data extraction date: March 28, 2021

Outcome: **Laninamivir** prescription

Variables: Age groups, sex, setting (admitted or outpatient), and hospital beds (20–299, 300–499, or ≥ 500)

|  | Multivariate analysis | | |
| --- | --- | --- | --- |
|  | Adjusted odds ratio (95% CI) | | P-value |
| **Age groups** |  |  |  |
| ≥ 65 | Ref |  |  |
| 12–64 | 2.210 | (2.140–2.280) | <0.001 |
| 6–11 | 2.140 | (2.060–2.230) | <0.001 |
| ≤5 | 0.091 | (0.083–0.100) | <0.001 |
| **Sex** |  |  |  |
| Male | Ref |  |  |
| Female | 1.030 | (1.010–1.050) | <0.001 |
| **Setting** |  |  |  |
| Outpatient | Ref |  |  |
| Inpatient | 0.246 | (0.230–0.263) | <0.001 |
| **Hospital beds** |  |  |  |
| 20–299 | Ref |  |  |
| 300–499 | 0.709 | (0.692–0.726) | <0.001 |
| ≥ 500 | 0.690 | (0.670–0.710) | <0.001 |

# Additional Table 5. Multivariate analysis of patient characteristics in 2018/2019 season

Data source: 2018-Nov, 2018-Dec, 2019-Jan, 2019-Feb, 2019-Mar

Data extraction date: March 28, 2021

Outcome: **Oseltamivir** prescription

Variables: Age groups, sex, setting (admitted or outpatient), and hospital beds (20–299, 300–499, or ≥ 500)

|  | Multivariate analysis | | |
| --- | --- | --- | --- |
|  | Adjusted odds ratio (95% CI) | | P-value |
| **Age groups** |  |  |  |
| ≥ 65 | Ref |  |  |
| 12–64 | 0.661 | (0.644–0.678) | <0.001 |
| 6–11 | 0.647 | (0.626–0.670) | <0.001 |
| ≤5 | 5.840 | (5.610–6.070) | <0.001 |
| **Sex** |  |  |  |
| Male | Ref |  |  |
| Female | 1.010 | (0.993–1.030) | 0.202 |
| **Setting** |  |  |  |
| Outpatient | Ref |  |  |
| Inpatient | 0.331 | (0.319–0.344) | <0.001 |
| **Hospital beds** |  |  |  |
| 20–299 | Ref |  |  |
| 300–499 | 1.580 | (1.550–1.620) | <0.001 |
| ≥ 500 | 1.640 | (1.600–1.680) | <0.001 |

# Additional Table 6. Multivariate analysis of patient characteristics in 2018/2019 season

Data source: 2018-Nov, 2018-Dec, 2019-Jan, 2019-Feb, 2019-Mar

Data extraction date: March 28, 2021

Outcome: **Peramivir** prescription

Variables: Age groups, sex, setting (admitted or outpatient), and hospital beds (20–299, 300–499, or ≥ 500)

|  | Multivariate analysis | | |
| --- | --- | --- | --- |
|  | Adjusted odds ratio (95% CI) | | P-value |
| **Age groups** |  |  |  |
| ≥ 65 | Ref |  |  |
| 12–64 | 0.205 | (0.193–0.218) | <0.001 |
| 6–11 | 0.521 | (0.483–0.562) | <0.001 |
| ≤5 | 0.848 | (0.800–0.899) | <0.001 |
| **Sex** |  |  |  |
| Male | Ref |  |  |
| Female | 0.960 | (0.920–1.000) | 0.063 |
| **Setting** |  |  |  |
| Outpatient | Ref |  |  |
| Inpatient | 27.500 | (26.300–28.800) | <0.001 |
| **Hospital beds** |  |  |  |
| 20–299 | Ref |  |  |
| 300–499 | 1.100 | (1.040–1.160) | <0.001 |
| ≥ 500 | 1.410 | (1.340–1.500) | <0.001 |

# Additional Table 7. Multivariate analysis of patient characteristics in 2018/2019 season

Data source: 2018-Nov, 2018-Dec, 2019-Jan, 2019-Feb, 2019-Mar

Data extraction date: March 28, 2021

Outcome: **Zanamivir** prescription

Variables: Age groups, sex, setting (admitted or outpatient), and hospital beds (20–299, 300–499, or ≥ 500)

|  | Multivariate analysis | | |
| --- | --- | --- | --- |
|  | Adjusted odds ratio (95% CI) | | P-value |
| **Age groups** |  |  |  |
| ≥ 65 | Ref |  |  |
| 12–64 | 3.880 | (3.310–4.540) | <0.001 |
| 6–11 | 11.900 | (10.100–14.000) | <0.001 |
| ≤5 | 0.690 | (0.541–0.880) | 0.003 |
| **Sex** |  |  |  |
| Male | Ref |  |  |
| Female | 1.100 | (1.030–1.170) | 0.004 |
| **Setting** |  |  |  |
| Outpatient | Ref |  |  |
| Inpatient | 0.209 | (0.155–0.282) | <0.001 |
| **Hospital beds** |  |  |  |
| 20–299 | Ref |  |  |
| 300–499 | 1.040 | (0.965–1.120) | 0.298 |
| ≥ 500 | 1.760 | (1.620–1.900) | <0.001 |

# Additional Figure 1. Baloxavir prescription rate from the 2018/2019 season to the 2019/2020 season


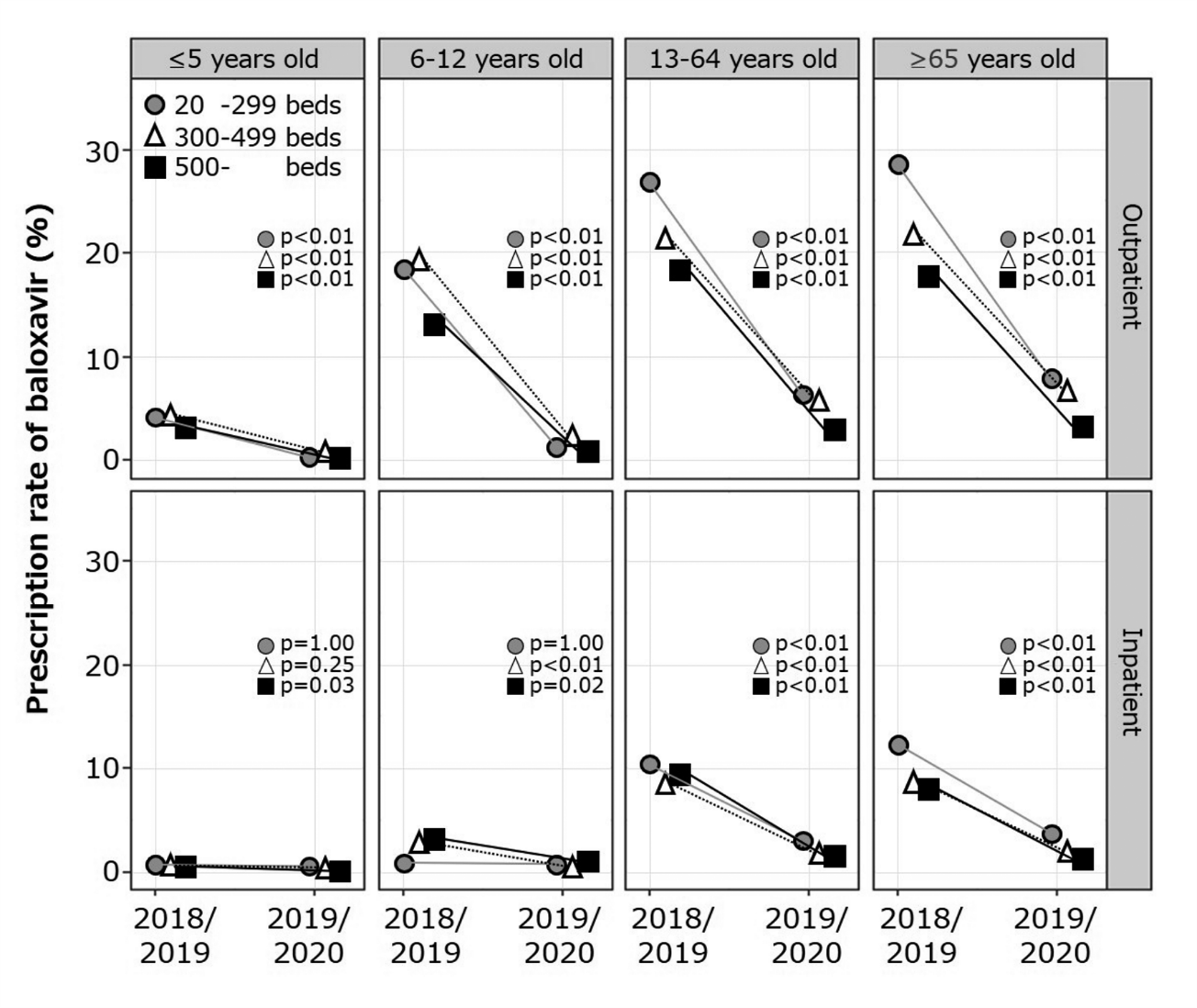


The rate of baloxavir prescription in patients who received anti-influenza drug was calculated for outpatient vs. inpatient settings, using the variables age group and hospital bed capacity. The difference in baloxavir prescription rates between the 2018/2019 and the 2019/2020 season was analyzed using Fisher’s exact test.
